# Supplementary material for: Mediterranean Diet in Older Adults: Cardiovascular Outcomes and Mortality from Observational and Interventional Studies—A Systematic Review and Meta-Analysis
Source: Nutrients. 2024 Nov 19;16(22):3947. doi: 10.3390/nu16223947 (PMC11597443; doi:10.3390/nu16223947)
Supplement: Supplementary file 1 [file nutrients-16-03947-s001.zip › nutrients-3228024-supplementary.pdf]

FIGURES

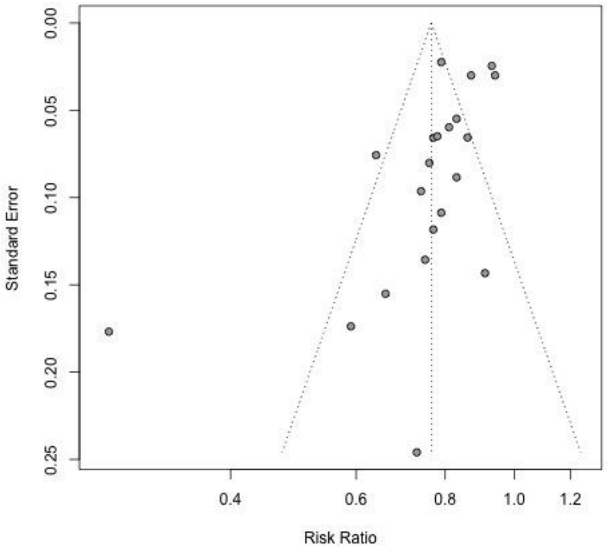

Figure S1. Funnel plot of studies on all-cause mortality outcome

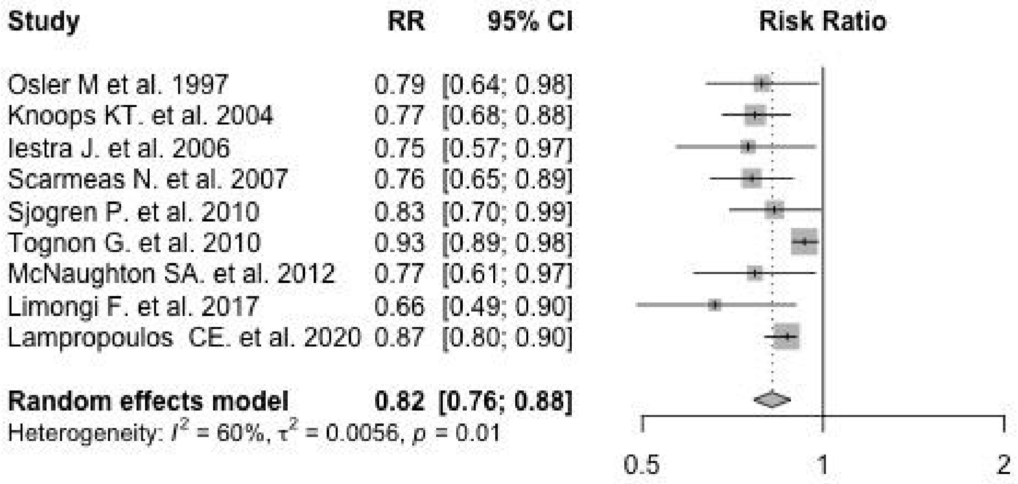

Figure S2. MD adherence and all-cause mortality over 70 years of age

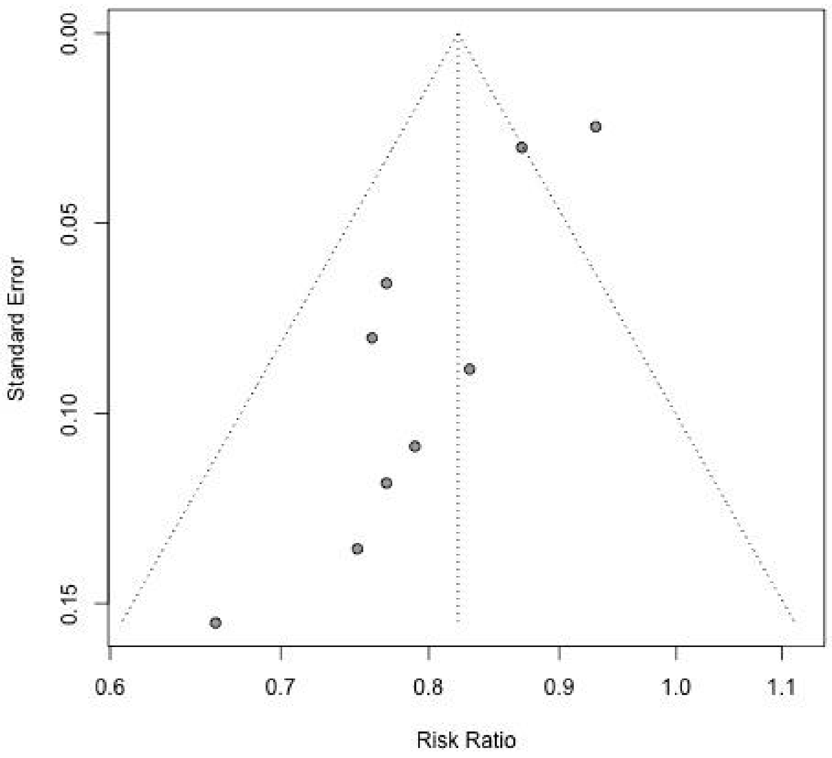

Figure S3. Funnel plot of studies on all-cause mortality outcome in subjects >70 years old

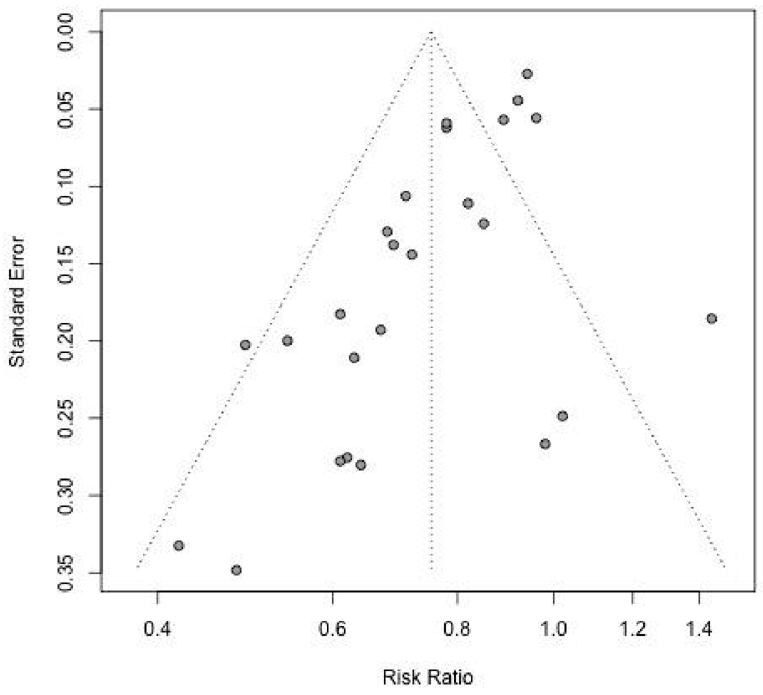

Figure S4. Funnel plot of studies on overall cardiovascular events

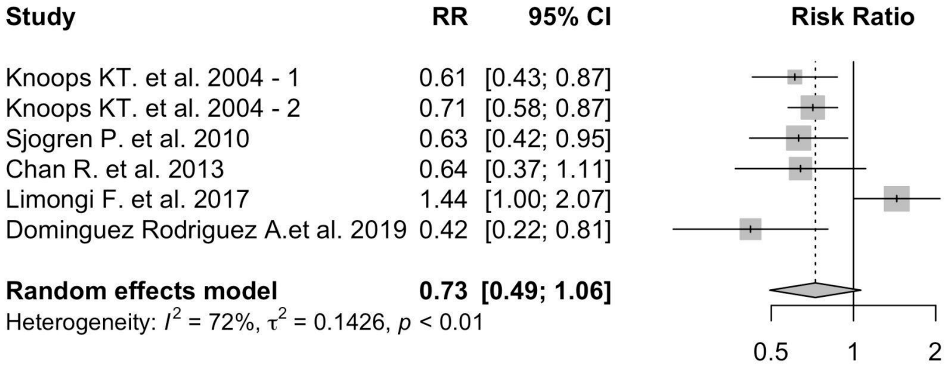

Figure S5. MD adherence and overall cardiovascular events over 70 years of age

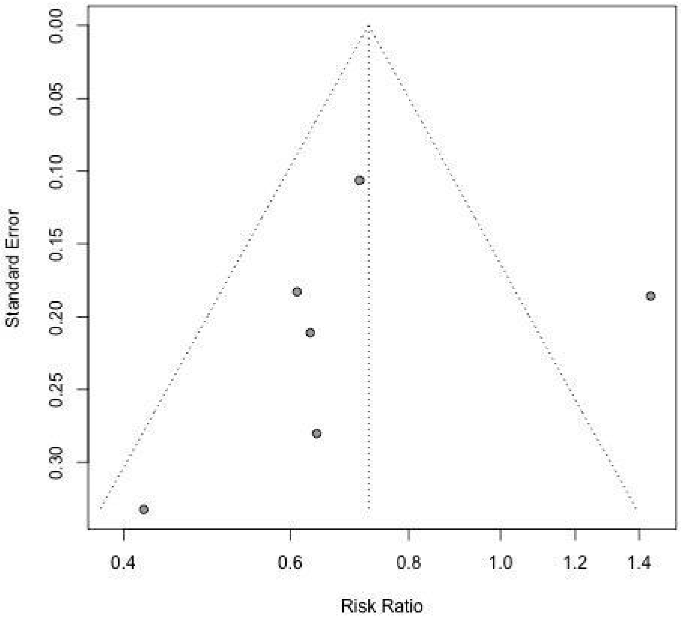

Figure S6. Funnel plot of studies on overall cardiovascular events in subjects >70 years old

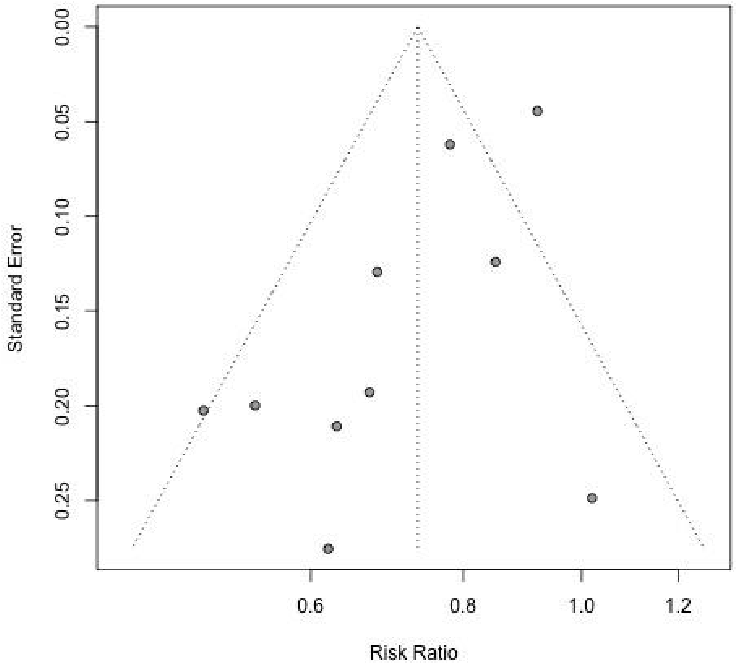

Figure S7. Funnel plot of studies on cardiovascular mortality

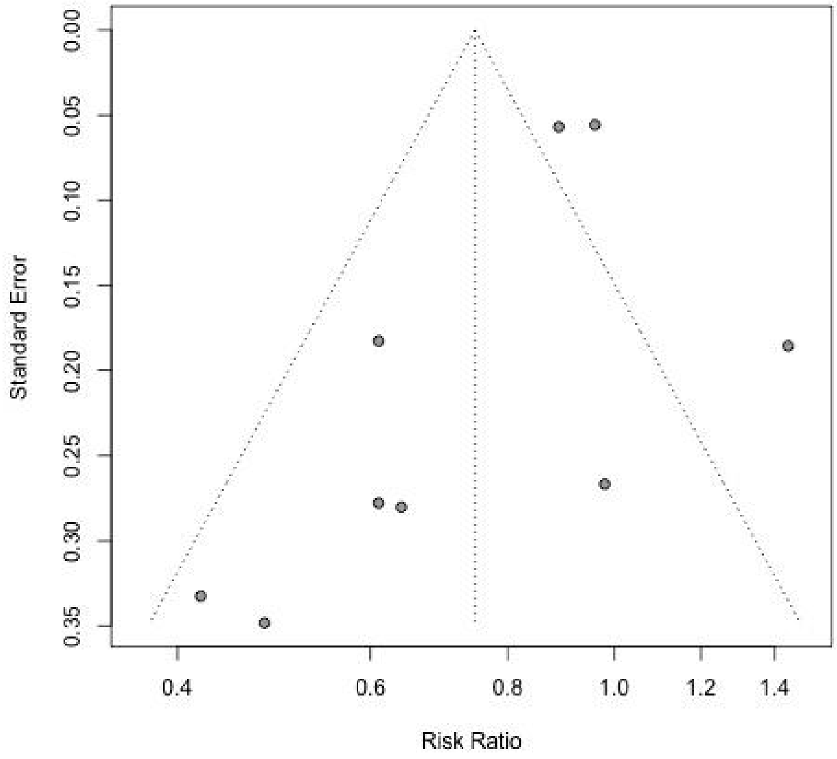

Figure S8. Funnel plot of studies on cardiovascular non-fatal events

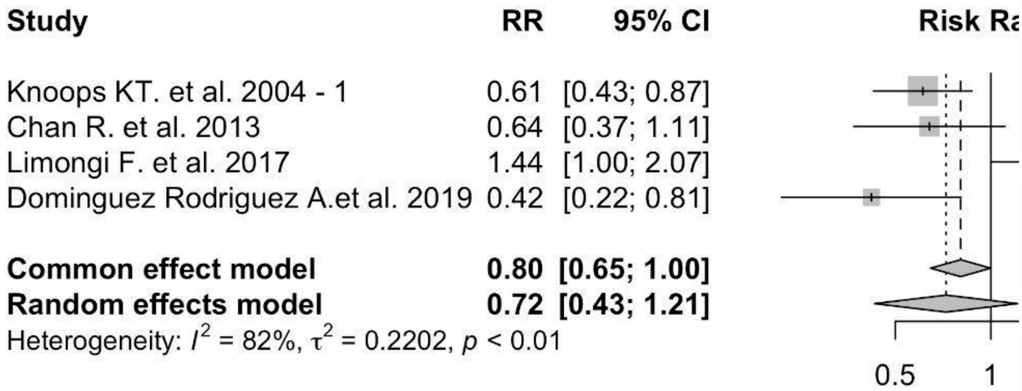

Figure S9. MD adherence and cardiovascular non-fatal events over 70 years of age

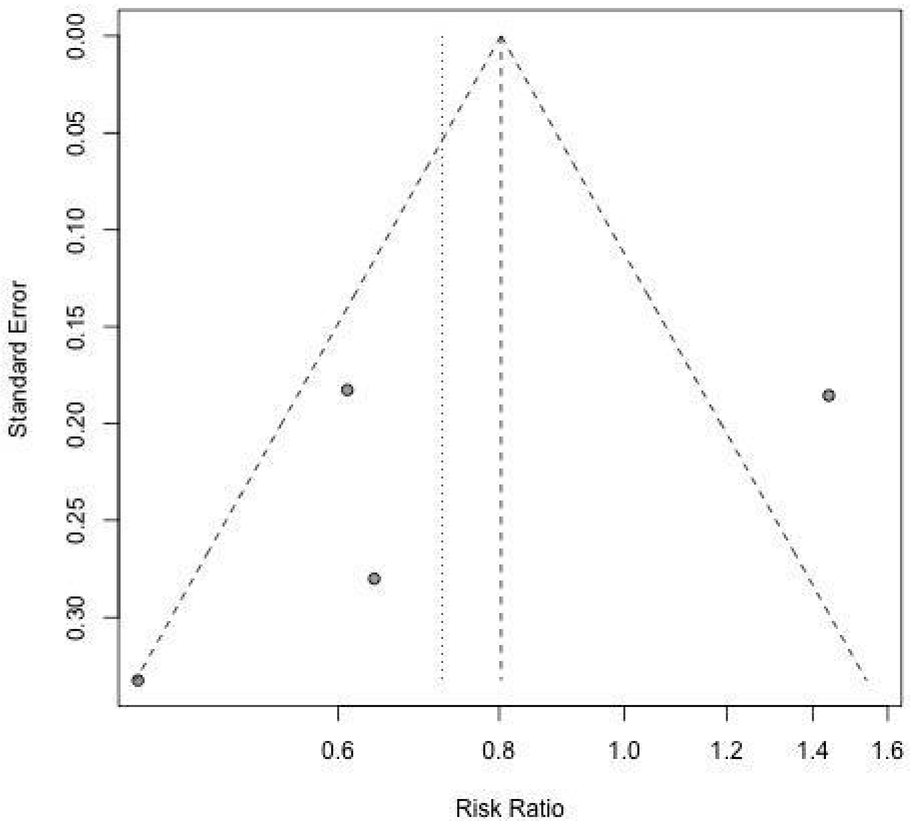

Figure S10. Funnel plot of studies on cardiovascular non-fatal events over 70 years of age

TABLES

Table S1. RoB2 risk assessment tool

Unique ID	Study ID	D1	D2	D3	D4	D5	Overall
1	Estruch R. et al, 2018	+	!	+	+	+	!
2	Papadaki et al, 2017	+	!	+	+	!	!

-	High risk
D2	Deviations from the intended interventions
D3	Missing outcome data
D4	Measurement of the outcome
D5	Selection of the reported result

Table S2. NOS risk assessment tool

Cohort studies	Selection (4)	Comparability (2)	Outcome (3)	Total score (9)	Risk
	**Newcastle-Ottawa Quality Assessment Scale**				
Limongi F. et al., 2017	***	**	**	7	Low
Sjogren P. et al., 2010	***	**	**	7	Low
Knoops KT. et al., 2004	***	**	**	7	Low
Iestra J. et al., 2006	**	**	***	7	Low
Lampropoulos CE. et al., 2020	***	*	**	6	Medium
Corley J., 2020	****	**	**	8	Low
Hershey MS. et al., 2020	***	**	**	7	Low
Tognon G. et al., 2010	***	**	**	7	Low
Trichopoulou A. et al., 2005	***	*	**	6	Medium
Bertoia ML. et al., 2014	**	**	**	6	Medium
Tognon G. et al., 2014	***	*	**	6	Medium
Mitrou PN. et al., 2007	***	*	**	6	Medium
Van Den Brandt, 2011	***	*	***	7	Low
Kouris-Blazos A. et al., 1999	*	*	*	3	High
Scarmeas N. et al., 2007	**	*	**	5	Medium
Lopez Garcia E. et al., 2014	*	*	***	5	Medium
McNaughton SA. et al., 2012	***	**	**	7	Low
Whalen KA. et al., 2017	***	**	***	8	Low
Trichopoulou A. et al., 2007	*	*	**	4	Medium
Dilis V. et al., 2012	**	*	***	6	Medium
Dominguez Rodriguez A. et al., 2019	**	*	**	5	Medium
Gardener H. et al. 2011	**	*	**	5	Medium

Lau KK. et al. 2015	***	**	***	8	Low
Chan R. et al. 2013	**	*	**	5	Medium
Cardenas Fuertes G. et al., 2018	***	*	**	6	Medium
Osler M. et al., Denmark, 1997	*	*	*	3	Low

0-3 points: high risk of bias; 4-6 points: medium risk of bias; 7-9 low risk of bias
follow up considered adequate if >1 year
